# Biogeographical and evolutionary importance of the European high mountain systems

**DOI:** 10.1186/1742-9994-6-9

**Published:** 2009-05-29

**Authors:** Thomas Schmitt

**Affiliations:** 1 Biogeographie, Fachbereich VI, Wissenschaftspark Trier-Petrisberg, Universität Trier, D – 54286 Trier, Germany

## Abstract

Europe is characterised by several high mountain systems dominating major parts of its area, and these structures have strongly influenced the evolution of taxa. For species now restricted to these high mountain systems, characteristic biogeographical patterns of differentiation exist. (i) Many local endemics are found in most of the European high mountain systems especially in the Alps and the more geographically peripheral regions of Europe. Populations isolated in these peripheral mountain ranges often have strongly differentiated endemic genetic lineages, which survived and evolved in the vicinity of these mountain areas over long time periods. (ii) Populations of taxa with wide distributions in the Alps often have two or more genetic lineages, which in some cases even have the status of cryptic species. In many cases, these lineages are the results of several centres of glacial survival in the perialpine areas. Similar patterns also apply to the other geographically extended European high mountain systems, especially the Pyrenees and Carpathians. (iii) Populations from adjoining high mountain systems often show similar genetic lineages, a phenomenon best explained by postglacial retreat to these mountains from one single differentiation centre between them. (iv) The populations of a number of species show gradients of genetic diversity from a genetically richer East to a poorer West. This might indicate better glacial survival conditions for this biogeographical group of species in the more eastern parts of Europe.

## Background

The evolution within species and sibling species complexes in Europe is closely connected to the range shifts caused by the cyclic changes between relatively short and warm, mostly humid and longer cold and predominantly dry periods [1,2]. These climatic shifts resulted in large scale range shifts in many species often resulting in disjunct distribution pattern during at least one of these phases [3,4]. However, the effects of these climatic fluctuations vary considerably in different ecological and distributional groups [5,6], and three major groups can be distinguished: Mediterranean, continental and arctic/alpine species [2], which are all to some degree influenced in their biogeography and evolution by the complexity of the European high mountain systems.

The Mediterranean species survived the cold stages of the Pleistocene in the Mediterranean Basin and expanded northwards during the postglacial [7,8]. These expansions are largely shaped by the high mountain systems of the Alps and Pyrenees acting as dispersal barriers of different strength so that four different paradigms of postglacial range expansions from the three Mediterranean peninsulas can be distinguished [9,10]. In contrast to the assumptions made by de Lattin [6], the continental species, which in most cases have not immigrated into Europe from Asiatic core areas during the postglacial, had so called extra-Mediterranean refuge and differentiation centres north of the typical Mediterranean refugia. These were also strongly influenced by the European mountain systems because they offered suitable refuge areas in their non-glaciated parts during ice ages and acted as dispersal barriers during postglacial range shifts [e.g. [11-19]].

The group of alpine and arctic-alpine species is most strongly shaped in their biogeographical and evolutionary history by the geographic location and complexity of the different European high mountain systems because (i) these species are now restricted to these mountain ranges and (in some cases) the arctic and (ii) most of their actual distribution areas were covered by extensive ice-shields during the glacial periods [5,20]. In general, we can distinguish between several different distributional types in these mountain species. Thus, Varga and Schmitt [20] distinguish between five major patterns: (i) endemics of the Alps restricted to some parts or the entire range, (ii) the "Alpine archipelago" with the species' strongholds in the Alps and other parts of the range in adjoining mountain ranges, but without long distance disjunctions, (iii) mountain species with long distance disjunctions in Europe (e.g. in the mountains of the Balkan Peninsula, Italy or Iberia), (iv) oro-Mediterranean species with their ranges mostly restricted to the mountains of the summer-arid high mountain systems of southern Iberia, southern Italy and the southern Balkan Peninsula and (v) arctic-alpine species with a disjunction between mountain populations in the South (Alps, often also Pyrenees, Carpathians, Balkan high mountain systems) and a large zonal range in the Arctic.

However, the distribution histories and evolutionary processes underlying these different distributional types in mountain species are still poorly understood. Therefore, I summarise the known examples and extract the repetitive pattern. Examples are preferably taken from the butterflies because this group is well studied and suitable for biogeographical analyses [e.g. [21-29]], but where necessary other animal groups and even plants are referenced. In this article, I especially focus on two questions:

(1) Which repetitive biogeographical and evolutionary patterns are characteristic for European mountain species?

(2) What is the influence of the climatic cycles on their differentiation?

## Endemics and endemic genetic lineages of high mountain systems

### Endemics of the Alps

Most of the larger European high mountain systems possess endemic species (or at least subspecies) confined to just one of them. As the Alps are by far the most extensive and highest mountain system of Europe, it is not surprising that the highest number and amount of endemics of typical high mountain species is found in this mountain range, as for example demonstrated by lepidopterans and especially in the little mobile group of the micro-moths [20]. Some of these alpine endemics (in animals as well as in plants) are widespread all over this mountain area, stretching almost the entire range from Nice in the southwest to Vienna in the northeast (in many cases showing remarkable genetic substructure, see below); while others are more or less local endemics of some parts of the Alps.

**Figure 1:**
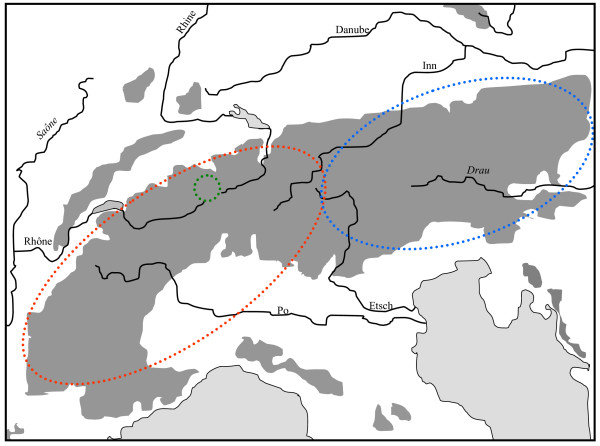
**Geographic distribution of three genetic lineages based on allozyme polymorphisms of the *Erebia melampus*/*sudetica *butterfly species complex in the Alps**. (Red: western Alpine *E. melampus*; blue: eastern Alpine *E. melampus*; green: *E. sudetica inalpina*). Redrawn from Haubrich & Schmitt [31].

Two types of hotspots for local endemics of the Alps can be distinguished:

(i) Species with their ranges restricted to peripheral regions of the Alps and mostly confined to lower and intermediate elevations (e.g. observed in some *Erebia *species, Lycaenids etc.; for details see Varga & Schmitt [20]). The largest concentrations of these local endemics are in the regions of the southwestern and the southeastern Alps, two regions with larger areas at lower altitudes not covered by ice during glaciations. These ice-free areas most probably served as centres of glacial survival, from where these species only performed altitudinal shifts, but no major range expansions over large parts of the deglaciating Alps. As these taxa might have performed these altitudinal shifts repeatedly through the climatic fluctuations of the Pleistocene, these refuge areas might also be the locations of the evolutionary process of speciation [1]. However, genetic analyses in this group of species are scarce and in butterflies restricted to the three taxa *Coenonympha darwiniana *[30], *C. macromma *[30] and *Erebia sudetica inalpina *(Figure 1) [31]. The latter is today confined to the region of Grindelwald (Berner Oberland, northwestern Alps) and most probably survived at least the last glaciation in a geographically restricted refuge area northwest of the Alpine ice-shield, likely suffering population bottlenecks and subsequent genetic erosion [31]. The two *Coenonympha *taxa are restricted to the south-central Alps (*C. darwiniana*) and the Alpes Maritimes (*C. macromma*). Both might have survived also at least the last ice age at lower elevations close to their recent distributions [30].

(ii) Species restricted to some parts of the Inner Alps, as for example a number of (often flightless) micro-moth species [32] and beetles [5], but only a single butterfly species (i.e. *Erebia nivalis*) [20]. These species in general are confined to high alpine habitats and may have survived glaciations *in situ *at so called Nunataks (i.e. small ice-free areas topping over the ice-shield of the Inner Alps). For animals, survival in these areas is only supported by chorological data sets (i.e. distribution pattern) [5,20], but genetic data in plants give some support for these refuges (see Schönswetter et al. [33] for a recent review, [34,35]).

### Endemics of other high mountain systems of Europe

Although the Alps are the European mountain system with the highest number of endemic species, numerous endemic genetic lineages or taxa are known for almost all high mountain systems in parts of Europe not entirely being covered by ice during the glacial periods. However, there are peculiar differences between the more marginal (e.g. Cantabrian Mts., Bulgarian Mts.) and more central (e.g. Carpathians, Massif Central, Apennines) mountain systems.

**Figure 2:**
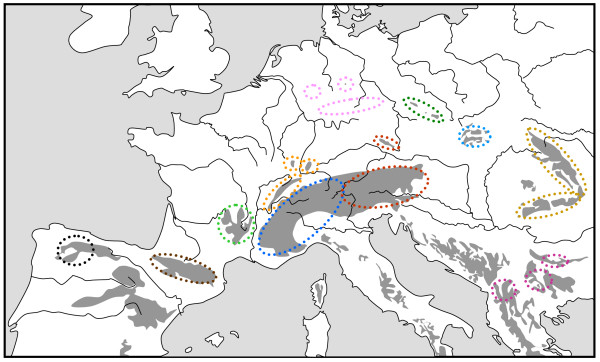
**Geographic distribution of twelve genetic lineages based on mtDNA sequences of the caddisfly *Drusus discolor *in the European mountain systems**. Each lineage is presented in a different colour; separate areas marked by identical colour harbour the same lineage. Redrawn from Pauls et al. [41].

Thus, the more marginal mountain areas of Europe have particularly high proportions of old evolutionary units (often with relict status) when compared to more geographically central mountain ranges. For example, cases of such old genetic lineages are known for the flora of the Cantabrian Mts. in northwestern Spain [e.g. [36-38]]; the number of examples in animals is rather low, but the known cases support the patterns observed in plants well, e.g. the butterfly *Proclossiana eunomia *[39,40] and the caddisfly *Drusus discolor *(Figure 2) [41].

**Figure 3:**
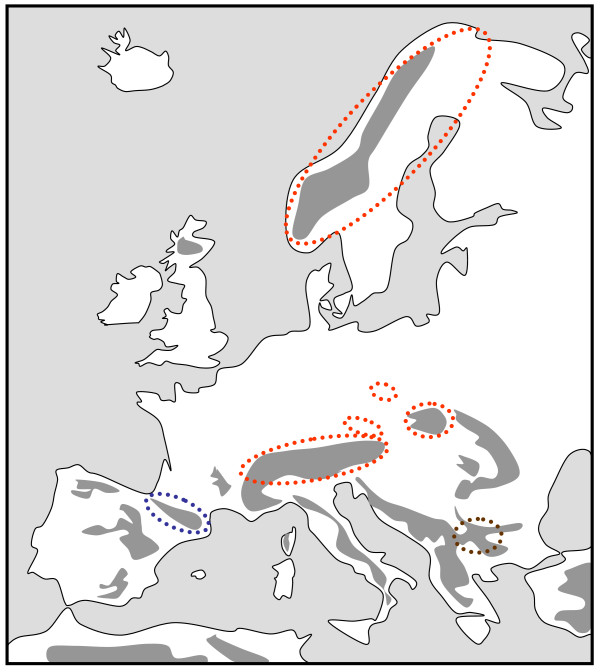
**Geographic distribution of three genetic lineages based on mtDNA sequences of the spider species complex *Pardosa saltuaria *in the European mountain systems and Scandinavia**. Each lineage is presented in a different colour; separate areas marked by identical colour harbour the same lineage. Redrawn from Muster & Berendonk [43].

Another stronghold of strongly differentiated genetic lineages and endemic species is situated in the Bulgarian high mountain systems. Thus, the most distinct genetic lineages of the beetle *Nebria rufescens *and the butterfly *Erebia pandrose*, two arctic-alpine species, were found here [42]; a strongly differentiated genetic lineage was also found in the *Proclossiana eunomia *populations from the Stara Planina [39]. In the spiders of the *Pardosa saltuaria *species group, the populations from the Bulgarian mountains, and also from the Pyrenees, showed a strong differentiation from a northern clade including the Alps, Carpathians, some middle high mountains of Central Europe and Scandinavia (Figure 3) [43]. The caddisfly *Drusus discolor *also showed strongly differentiated genetic lineages for all of the marginal mountain systems of Europe where the taxon occurs, including the high mountain systems of Bulgaria (Figure 2) [41]. Also plants in the Balkan high mountain systems showed strongly differentiated genetic lineages from other regions of Europe [44].

Furthermore, the distribution pattern of some species and subspecies strongly support these observed genetic structures. Thus, the eastern Balkan mountains are the only mountain range of the southern part of Europe where the fairly widespread mountain ringlet *Erebia epiphron *is lacking, but the species is substituted by its sister taxon *Erebia orientalis *supporting the strong evolutionary independence of this region. Regarding this latter species, each of the three mountain ranges harbouring it (i.e. Rila, Pirin, Stara Planina) has its own subspecies with strong morphological differentiations among them [45,46]. A similar situation is also observed for the only occurrences of *Euphydryas cynthia *outside the Alps: the male individuals of the Pirin population consistently have a considerably more extended white wing colouration than average populations from the Alps, while the white colouration is strongly reduced in the Rila individuals [45]. This also underlines the evolutionary independence among these mountain ranges of the eastern Balkan Peninsula on a fairly small geographic scale of sometimes only few tens of kilometres.

Such old lineages are fewer in the mountain systems with closer geographic connections with the Alps (e.g. Carpathians, Massif Central), and genetic differentiations of these mountain systems from the Alps are often low; most examples come from plants [e.g. [36,38,47,48]], but also include the *Pardosa saltuaria *spider species group (Figure 3) [43]. On the species level, these mountain ranges mostly share their species with the Alps. However, well distinguished subspecies are known for these mountain species with many examples in the genus *Erebia *(e.g. *E. epiphron*, *E. euryale*, *E. gorge*, *E. manto*, *E. melas*) [49], but the ages of these splits are mostly unknown and some even might be rather recent, even postglacial.

**Figure 4:**
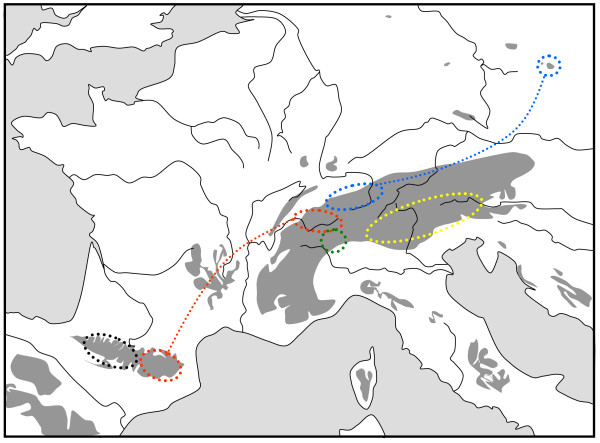
**Geographic distribution of five genetic lineages based on allozyme polymorphisms of the butterfly *Erebia epiphron *in the western European mountain systems**. Each lineage is presented in a different colour; separate areas marked by identical colour harbour the same lineage and are linked by a dotted line in the same colour. Redrawn from Schmitt et al. [50].

**Figure 5:**
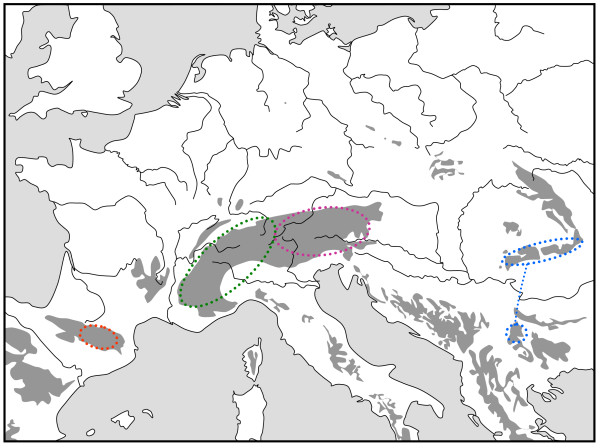
**Geographic distribution of four genetic lineages based on allozyme polymorphisms of the butterfly *Erebia euryale *in the European mountain systems**. Each lineage is presented in a different colour; separate areas marked by identical colour harbour the same lineage and are linked by a dotted line in the same colour. Redrawn from Schmitt & Haubrich [51].

The Pyrenees have an intermediate position, in which they show some close connections to the Alps (see below), but also have numerous endemic species (e.g. in lepidopterans *Erebia gorgone*, three *Sattleria *species) [20,49]; their number however is much lower than in the Alps, which are considerably higher and much more extended. Many mountain species with larger distributions show endemic lineages in the Pyrenees or parts of them, thereby supporting the idea of differentiation centres at the lower elevations of the Pyrenean area and recent (mostly postglacial) range shifts to higher altitudes of the mountains, as demonstrated for the butterflies *Erebia epiphron *(Figure 4) [50], *Erebia euryale *(Figure 5) [51], *Erebia pandrose *[42] and *Erebia manto *(unpublished data), but also other animal [41,43] and plant species [36].

The summer-arid high mountain areas of the extreme south of Europe (especially southern Iberia and the southern Balkan Peninsula) and the Maghreb (here in particular the Atlas Mts.) do not have the typical alpine zonation [52], but show typical oro-Mediterranean characteristics. Some mountain species show local endemism to strongly confined mountain blocks in this area as e.g. known for a number of butterfly species [20,45,49]. These species in many cases represent relicts of often old invasions of species with their closed biogeographical connections to Central Asia [20,53,54].

## Different genetic lineages within one high mountain system

The large high mountain systems of the southern part of Europe (i.e. Pyrenees, Alps and Carpathians) have in many cases been colonised after the postglacial deglaciation by several genetic lineages, which have survived at least the last glacial period in several allopatric refugial areas often adjoining these high mountain systems.

### Different genetic lineages in the Alps

The Alps as the largest European high mountain system show a remarkable phylogeographic structure in many species. In plants, a differentiation into four genetic units along the Alps seems the most common feature with these lineages typically being confined to the southwestern, western central, eastern central and eastern Alps and the eastern lineages more often being of higher genetic diversity than the more western ones [2,33,42]. A quite similar pattern to that in many plant species was proposed for the leaf beetle *Oreina elongata *[55]. Also other animals frequently show different genetic lineages in the Alps, but their number is often less than in plants, and in many cases, only a western and an eastern lineage can be distinguished. This is the case in the two butterflies *Erebia melampus *(Figure 1) [31] and *Erebia euryale *(Figure 5) [51], the wolf spiders species group *Pardosa saltuaria *(Figure 3) [43] and the caddisfly *Drusus discolor *(Figure 2) [41]. In the first case, two strongly differentiated lineages (supported by allozyme polymorphisms [31] and differences in the male genitalia [56]) can be distinguished with a contact zone running north-south through Tyrol and along the Adige/Etsch valley (maybe with some hybridisation in a limited area of southeastern Switzerland and South Tyrol). The differentiation between these two lineages is rather advanced, and they most probably represent different species, with the eastern lineage showing a significantly higher genetic diversity than the western one [31]. These genetic and morphological differences are best explained by allopatric differentiation in two centres; and as the beginning of the first split is assumed to be older than the last ice age, the location of the initial differentiation centres must remain enigmatic. However, the most likely Würm glacial refuge centres are in the hilly areas south of the Upper Italian Lakes for the western lineage and in the lower elevation areas of the southeastern Alps (eastern Carinthia, Styria, Friul, parts of Slovenia) for the eastern lineage. Based on the higher genetic diversities of the populations belonging to the eastern lineage, we may assume that the survival conditions were more suitable in this eastern region and/or this refuge area was geographically considerably more extended than the more western refuge [31]. A very similar pattern was observed for *Erebia euryale *(Figure 5) with the only difference that survival of the western lineage is more likely around the southwestern Alps [51]. The caddisfly *Drusus discolor *repeats this geographic structure of a western and an eastern Alpine lineage (Figure 2) [41]. A much more complicated case is known in the butterfly *Erebia epiphron *[50]. This species comprises four strongly differentiated lineages in the Alps: western Alps, Aosta valley, northern Alps and eastern Alps (Figure 4). These lineages most probably evolved in four differentiation centres west, south, southeast and north of the Alps, often linking the Alps with other mountain systems (see below).

### Different genetic lineages in the Pyrenees

As in the Alps, more than one genetic lineage per species is also observed in mountain elements of the Pyrenees [20]. However, the Pyrenees are considerably less extended than the Alps, and the maximum number of different lineages found here is not as high as in the Alps; many species do not show marked differentiation over the Pyrenees. As an example, two different lineages are observed in the Pyrenees for *Erebia epiphron *(Figure 4), one in the western and the other in the eastern parts of these mountains, with the first one most probably surviving the last ice age north and the second in the southeastern parts of the Pyrenees [50]. Also for the plant *Cardamine alpina*, AFLP patterns suggest survival in multiple Pyrenean refugia [57].

### Different genetic lineages in the Carpathians

Although being the largest high mountain system of eastern/southeastern Europe, only a few genetic analyses include populations from more than one part of the Carpathian arc. However, compiling existing data for different plant species revealed a generally lower genetic diversity of the populations in the Carpathians than in the Alps, maybe due to higher topographic isolation of alpine habitats in the Carpathians [58].

One study on animals that included a considerable number of samples from the Carpathian region was performed on the caddisfly *Drusus discolor *and showed a remarkable differentiation between the northwestern and the southern Carpathians (Figure 2). This thus supports the idea of a long lasting separation between these two parts of this high mountain system predating at least the last glaciation [41]. Similar cases are also reported for plants as *Hypochaeris uniflora *[59] and *Campanula alpina *[60].

Thus, multiple glacial survival centres exist along the Carpathian arc, as in the cases of the Pyrenees and especially Alps, but the number of known cases is still rather limited. Therefore, a research focus on the phylogeography of the Carpathians is needed.

## Genetic links between different high mountain systems

As many of the glacial differentiation centres were not restricted to the foot-hills of the respective high mountain systems, but were located in the (hilly) areas between high mountain systems, many links (by species and genetic lineages) can be observed between adjoining high mountain systems, especially between (i) Pyrenees and southwestern Alps, (ii) northeastern Alps and northwestern Carpathians, (iii) southeastern Alps and western Balkan mountains and (iv) southern Carpathians and the eastern Balkan high mountain systems [20]. In the following, these connections are discussed using examples.

### Links between Pyrenees and southwestern Alps

The biogeographical links between the Pyrenees and the Alps are seen in the occurrences of many taxa with restricted distributions in these two areas, e.g. in lepidopterans by taxa as *Euchloe simplonia*, *Polyommatus g. glandon*, *Aricia n. nicias*, *Erebia o. oeme *[49]. However, the same genetic lineages are also repeatedly found in these two regions, as in the case of *Erebia cassioides *[61] and *Erebia epiphron *(Figure 4), which has genetically very similar populations in the western Pyrenees and the southwestern Alps, e.g. the Wallis in Switzerland [50]. The genetic similarity in this case is best explained by a Würm glacial link between the Pyrenees and the Alps, and thus a more widespread distribution in the hilly regions of France between these two high mountain systems during this time period. The postglacial disjunction has not been sufficiently long for the evolution of a clear differentiation between these now disjunct parts of the distribution. Similar patterns are also known for mountain plant species [47,62-66]. In the case of *Carex curvula*, the Pyrenean populations are nested within the western Alps group and show a low level of genetic diversity, probably due to recent long distance colonisation [67].

### Links between northeastern Alps and northwestern Carpathians

As in the case of the Pyrenees-Alps connections, many biogeographical interactions exist between the northeastern Alps and the northwestern Carpathians (i.e. Tatra Mts.). Typical examples are found in distribution patterns of lepidopterans like *Pieris bryoniae *and *Erebia pharte *[49]. An mtDNA analysis of the wolf spider species complex *Pardosa saltuaria *also underlines this link as the same mtDNA lineage was detected in the Alps and Carpathians (Figure 3) [43].

So far other genetic data for animals are lacking, but data on plants support this biogeographical link (e.g. for *Pritzelago alpina *[36] and *Senecio carniolicus *[48]). For *Ranunculus alpestris*, the Carpathians have been colonised stepwise from an eastern Alpine lineage [68], and the arctic-alpine *Ranunculus pygmaeus *colonised the Alps relatively recently from the east through the Tatra Mts. [69]. A relatively similar genetic link was observed between the northern Alps and the mountains of northern Moravia about 200 km west of the High Tatra Mts. for the butterfly *Erebia epiphron *(Figure 4) [50].

Nevertheless, many examples also revealed long-lasting separations between the populations from the Alps and the Carpathians [57,60,65,70].

### Links between southeastern Alps and western Balkan mountains

The southeastern edge of the Alps is biogeographically strongly linked with the mountains of the western Balkan Peninsula, as demonstrated by many occurrences of identical mountain beetle species in the southeastern Alps and the northwestern Balkan mountains [5,20]. While less in number, this distribution type is also known in the Lepidoptera for taxa like *Coenonympha gardetta*, *Erebia ottomana*, *E. stirius*, *E. oeme spodia*, *E. epiphron aetheria *and *E. styx trentae *[49]. These distribution patterns support the survival of mountain species in the southeastern parts of the Alps and adjoining areas (as demonstrated for *Erebia melampus *(Figure 1) [31] and *Erebia euryale *(Figure 5) [51]), with postglacial retreat into the Alps in the northwest and the northwestern Balkan mountains in the southeast.

Genetic data sets supporting these biogeographical links for animals are (to my knowledge) not available at the moment, but genetic data on plants support these connections e.g. in the species *Pulsatilla vernalis *[65] and *Polygonatum verticillatum *[38]. Considering morphological studies, the western Balkan populations of *Erebia pandrose *in the high mountain systems of Monte Negro, Serbia and Macedonia are closely related with populations existing in the southeastern Alps. This thus supports a recent (i.e. Würm glacial) biogeographical link between these two areas that are now separated by several hundreds of kilometres with no mountains over 2,000 m. On the contrary, the *Erebia pandrose *populations of the high mountain systems Rila and Pirin in Bulgaria are strongly differentiated from this group, and more closely related with the populations from the southern Carpathians [71], thus underlining a biogeographical east-west split throughout the central Balkan Peninsula [72].

### Links between southern Carpathians and eastern Balkan mountains

The Danube valley apparently has often acted as effective dispersal barrier between the Carpathians and the high mountain systems of the eastern Balkan Peninsula [20] with *Erebia medusa *being a well studied case in the Lepidoptera [18]. However, many connections between the mountain systems of these two areas are also known. Thus, a morphometric study of the male genitalia of *Erebia pandrose *demonstrated high similarities between these two high mountain areas [71] and hereby also supported a close biogeographical link between them.

An allozyme analysis of the mountain forest species *Erebia euryale *revealed by far the highest genetic diversity in this species in the populations from the Bulgarian Rila and the Romanian southern Carpathians, but no significant differentiation was detectable between these two areas (Figure 5) [51]. This is most probably due to the fact that these two areas were linked by cool forested areas during at least the last ice age allowing massive gene flow between these two regions; thus, at least the region of the Iron Gate (passage of the Danube through the southern Carpathians) must have been extensively covered by this type of forest during this time period. Furthermore, this study supports the conclusion of other analyses based on fossil remains (e.g. pollen, remains of animals) [73-78] that southeastern Europe was the most important glacial retreat of the representatives of the European mountain forest biome.

Furthermore, the distribution of many mountain species (e.g. *Erebia melas*, *E. cassioides*, *Coenonympha rhodopensis*) in the Bulgarian high mountain systems and the southern Carpathians underlines the biogeographical connections of the high mountain biota of both areas [20]. Genetic links between both areas are also known for plant species [67].

## Genetic links between high mountain systems and the Arctic

Recent (i.e. late glacial or postglacial) genetic links between the high mountain systems in the South and the Arctic in the North are a rather common biogeographical characteristic of many species with typical arctic-alpine disjunctions as already postulated by Holdhaus in his landmark book in 1954 [5]. This proposal is largely supported by many genetic analyses on plants [65,66,70,79-83] and animals (Figure 3) [42,43]. This argues for the wide distribution of these species in the periglacial steppes between the northern glacier and the glaciated mountains in the South and postglacial retreat to higher altitudes in the South, and higher latitudes in the North.

In butterflies, the mtDNA sequences of *Erebia pandrose *indicate a very close genetic connection between the populations from the Alps, Pyrenees and Scandinavia thus supporting their recent (i.e. postglacial) disjunction. However, the individuals from the Rila in Bulgaria show a stronger genetic differentiation from these other regions, thus supporting the idea that the Balkan populations were not included in this large zonal distribution during the last glaciation, but survived in an area of southeastern Europe not linked to the main distribution [42]. A similar structure was also observed in the leaf beetle *Nebria rufescens*, but the differences against the Balkan haplotypes were even stronger in this case [42]. Another butterfly study with the lycaenid *Aricia artaxerxes *revealed a very weak genetic differentiation between the populations from the northern UK and Scandinavia of only one or two mutations in the mtDNA cytochrome-b gene thus supporting the idea of a common source and only postglacial isolation [84]. A rather similar genetic structure was observed for the diving beetle *Hydroporus glabriusculus *[85]. The mountain hare *Lepus timidus *showed genetically very similar populations in the Alps and in Fennoscandia analysed by allozymes [86] microsatellites and mtDNA [87] underlining a postglacial common source for both regions between the Alpine and the Fennoscandian ice-shields. The lack of genetic differentiation between the Alps and Pyrenees in the arctic-alpine burnet *Zygaena exulans *also supports the idea of a survival of this species in a large zonal distribution range in the periglacial area of Europe during the last ice age and postglacial retraction to the high mountain systems and the Arctic [88].

## Genetic connections of middle high mountain systems

The middle high mountain systems of Europe show numerous biogeographical connections with the high mountain systems and among each other. Thus, the French Massif Central shows strong connections with the Pyrenees in many cases, e.g. by having the same melanistic subspecies *Erebia manto constans *[49] as well as showing rather weak genetic differentiation in *Parnassius apollo *[89] and *Pulsatilla vernalis *[65]. However, in other cases, close connections exist between the Massif Central and the Alps, as in the plant *Polygonatum verticillatum *[38]. However, the populations of the Massif Central are detected as even an independent phylogeographic group, as in the case of the caddisfly *Drusus discolor *(Figure 2) [41].

Links with the Alps are known for the Bavarian and Bohemian Forest, e.g. by sharing the eastern Alpine lineage in the caddisfly *Drusus discolor *(Figure 2) [41], and for the Jesenik mountains in northern Moravia, e.g. by striking genetic similarities with northern Alps *E. epiphron *populations (Figure 4) [50]. Relicts of alpine species in the Vosges and (to a lesser degree) in the Black Forest (e.g. in butterflies *Erebia epiphron*, *E. manto*, *Clossiana titania*, *C. thore*) [49] underline their close biogeographical relation with the Alps, which is also supported by genetic studies e.g. in the plant *Polygonatum verticillatum *[38]. In *Pulsatilla vernalis*, Tatra and Sudeten Mts. share the same chloroplast haplotype indicating a close link between these two mountain ranges for this species, but isolation from the Alps [65]. These patterns are most likely due to glacial refuge areas in the vicinity of the Alps, Carpathians or Pyrenees and postglacial retreat into one of these high mountain systems, but also into at least one of the adjoining middle high mountain systems with not sufficient time for differentiation since then.

**Figure 6:**
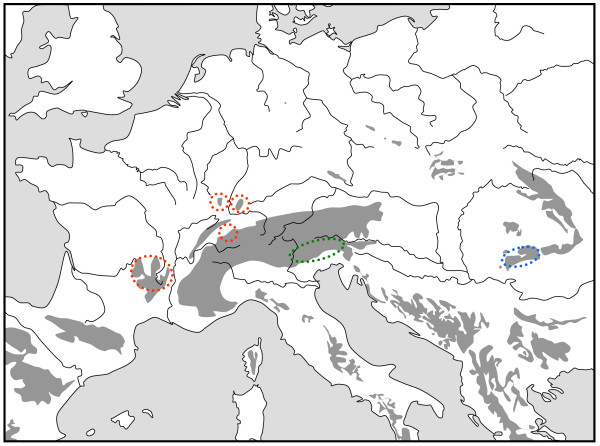
**Geographic distribution of three lineages based on morphometrics of the male genitalia of the caddisfly *Rhyacophila aquitanica *in the European mountain systems**. Each lineage is presented in a different colour; separate areas marked by identical colour harbour the same lineage. Redrawn from Bálint et al. [90].

Close biogeographical connections were also revealed among these middle high mountain systems. One striking example is the caddisfly *Drusus discolor *showing the same mtDNA lineage in the COI gene in middle high mountain systems (Figure 2) [41]. A similar pattern was also obtained in a morphometric study of another caddisfly species *Rhyacophila aquitanica *with very similar populations in the French Massif Central, the Vosges, the Black Forest and the Swiss canton of Fribourg (Figure 6) [90]. These patterns most probably result from a glacial distribution in the lower altitudes between these middle high mountain areas and postglacial retreat into the higher altitudes of these adjoining mountains.

## Conclusion

The topology of the European mountain systems had a strong influence on the biogeography and evolution of mountain and high latitude species. Due to the dynamic changes of these biota over time, a large variety of different evolutionary processes are responsible for the development of numerous biogeographical patterns. The most simple of these is the evolution of one genetic lineage or species endemic to one single mountain system due to vicariance with subsequent long processes of geographic isolation and evolution, as observed in almost all European high mountain systems. Especially in the Alps, but also in smaller high mountain systems, different genetic lineages exist beside each other as a result of evolution in geographically isolated areas, with often secondary contacts in these mountain systems. Where such centres of evolution and differentiation of lineages were located between two mountain systems, retraction during the postglacial into both of them is commonly resulting in one genetic lineage being found in two different mountain systems.

## Competing interests

The author declares that he has no competing interests.
